# Fabrication of Ion-Crosslinking Aminochitosan Nanoparticles for Encapsulation and Slow Release of Curcumin

**DOI:** 10.3390/pharmaceutics11110584

**Published:** 2019-11-07

**Authors:** Xiaoxiao Sun, Dongyan Yu, Zhuoyang Ying, Chuqiao Pan, Nan Wang, Fangfang Huang, Junhong Ling, Xiao-kun Ouyang

**Affiliations:** School of Food and Pharmacy, Zhejiang Ocean University, Zhoushan 316022, China; idsxx799@163.com (X.S.); dongyanyuzjou@163.com (D.Y.); zhuoyangyingzjou@163.com (Z.Y.); cqpanzjou@163.com (C.P.); ynwangnan@163.com (N.W.); gracegang@126.com (F.H.)

**Keywords:** aminated chitosan, folic acid, nanoparticles, targeted delivery, curcumin

## Abstract

Curcumin (Cur) has anticancer activities but has poor stability, which can be improved using carrier materials. In this study, chitosan was aminated to increase the number of amino groups on its surface, modified with folic acid (FA), and then made into nanoparticles by ionic crosslinking. Owing to ion interaction, the negatively charged, non-toxic tripolyphosphate (TPP) interacted with the positively charged amino group on the aminated chitosan (AmCS) surface, producing FA-AmCS-TPP nanoparticles, which were then characterized by scanning electron microscopy (SEM), transmission electron microscopy (TEM), Fourier transform infrared spectrophotometry (FT-IR), and thermogravimetric analysis (TGA). Their small particle size (175.2 ± 0.99 nm) and good surface positive potential (+42.4 mV) are beneficial for carrying antitumor drugs. We subsequently investigated whether coating of Cur by AmCS allows slow drug release by FA-AmCS-TPP nanoparticles in different pH environments, and estimated the Cur loading efficiency (EE-Cur). Our results showed that the cumulative release rate of Cur at 48 h was 56.2%, and that the EE-Cur reached 94.26 ± 0.91% with nanoparticles composed of 0.10 g AmCS, 10.0 mg FA, 10.0 mg TPP, and 15.0 mg Cur. Additionally, cytotoxicity experiments showed that the Cur/FA-AmCS-TPP nanoparticles had good targeting ability for tumor cells. Therefore, the non-toxic targeted composite nanoparticles had potential as a new antitumor agent that can overcome the limitations of Cur.

## 1. Introduction

Targeting drug delivery system (TDDS) is a drug delivery system in which a carrier is used to deliver drugs selectively to a targeted lesion site [1,2]. This system can reduce the adverse reactions, improve the efficacy, and reduce the dosage of drugs needed to achieve the desired effect, as well as to reduce the toxic and side effects of drugs to normal tissues. TDDS is not only the hotspot of pharmacy research but also a new method of tumor treatment that has attracted significant attention among researchers. Drug carrier materials and preparation techniques are the key to TDDS.

Chitosan, the main derivative of chitin, is the only natural alkaline polymorph in nature [3,4] owing to its abundant amino and hydroxyl groups on the surface [5,6]. Chitosan is an excellent carrier material [7,8,9] because of its good histocompatibility, diversity of biological activities, and absorption of degradation products (glucosamine) in vivo [10]. Chemical modification of chitosan can further improve its functionality [11,12,13,14] (mucosal adhesion, antimicrobial activity, and blood compatibility), such as alkylation, carboxymethylation, or quaternization.

Folate (FA), which is non-toxic and has weak immunogenicity, can specifically interact with folate receptors on the cell surface [15,16] to form complexes, and then enter the cell through endocytosis [17]. Active target agents constructed of FA showed good potential as antitumor agents and immunotherapies [18,19] because of their strong targeting effect [20], low toxicity [21], and ability to protect target molecules from degradation [22,23,24]. Chitosan can be used to modify FA on the surface of amino acids [25], which can be used to target excellent carrier materials. However, considering the following factors, the targeted composite must be further optimized: (i) chitosan beads and chitosan-inorganic composites [26,27], which are used for adsorption of metal ions, are usually prepared by crosslinking the amine groups on chitosan with glutaraldehyde [11]. The chemical residue during preparation raises the risk for biotoxicity in the body. In other words, a non-toxic cross-linking agent is needed to prepare chitosan particles, and sodium tripolyphosphate (TPP) stands out; (ii) compared to the traditional micron-sized carrier materials used in the delivery process, it is generally believed [28] that nano-sized carrier currently possess quite good performance [29,30,31] (large specific surface area, stronger targeting, and increased drug bioavailability); (iii) surface particles with higher positive charge tend to attract negatively charged particles on the cell membrane [12,32], which is beneficial to the distribution of particles in the body after drug administration, and is of great significance in the treatment of solid tumors; thus, it was suggested that these nanoparticles have great potential for targeting tumors.

In addition, curcumin (Cur) has blood lipid-lowering, anticoagulant, antioxidant, and anticancer activities, as well as a wide range of clinical applications [33,34,35]. However, it is sensitive to light, heat, and iron ions. Therefore, it is necessary to improve the stability of Cur through preparation using carrier materials [36,37,38,39,40].

Based on the above research background, for tumor targeting, chitosan was aminated (AmCS) to increase its surface positive charge [41] and FA was modified to achieve a stronger targeting effect [42]. In addition, the non-toxic and non-biological risk FA-AmCS-TPP nanoparticles prepared by simple ion crosslinking has great potential for carrying such fat-soluble drugs as Cur, and are of great significance for the targeted treatment of solid tumors. The resulting FA-AmCS-TPP nanoparticles were then characterized by scanning electron microscopy (SEM), transmission electron microscopy (TEM), Fourier-transform infrared spectrophotometry (FT-IR), differential scanning calorimetry (DSC) and thermogravimetric analysis (TGA). The characteristics of the nanoparticles, including their particle size, polydispersity index (PDI), and zeta potential, were compared between different compositions. In addition, the non-toxic and stable nanoparticles generated by TPP cross-linking are expected to encapsulate drugs well. Coating of a drug with AmCS to form AmCS-TPP composite nanoparticles [43] may generate strong mechanical resistance to protect the drug from oxidation and deterioration [44,45,46]. Thus, we evaluated the practical application prospect of FA-AmCS-TPP composite nanoparticles by investigating the loading rate and release rate of Cur, as a model drug, from the solid nanoparticles under different pH environments. Furthermore, cytotoxicity and uptake by tumor cells were also assessed to evaluate the targeting effect of the FA-AmCS-TPP nanoparticle water dispersion systems.

## 2. Materials and Methods 

### 2.1. Materials

Chitin, chitosan (deacetylation degree: 95%, viscosity: 100–200 mPa·s), hydrogen peroxide (purity of 30%), disodium hydrogen phosphate, sodium dihydrogen phosphate, folic acid (FA; purity of ≥98%), sodium tripolyphosphate (TPP), curcumin (Cur), sodium hydroxide (NaOH), acetic acid, NH_3_·H_2_O (25 wt %), p-benzoquinone (p-BQ), ethylenediamine (EDA), dimethyl sulfoxide (DMSO), coumarin-6, and hydrochloric acid were purchased from Aladdin Chemical Co., Ltd. (Shanghai, China). All these reagents and chemicals were of analytical grade and used without special treatment. Human colon cancer cell line (LS174T) was purchased from Cell Bank of Chinese Academy of Sciences (Shanghai, China).

### 2.2. Preparation of Aminated Chitosan (AmCS) 

AmCS was prepared using a previously published method with a few modifications [47]. The preparation process was divided into three steps. First, 0.50 g of chitin was soaked in 100 mL p-BQ solution (4 mM), which is a coupling agent, and the pH was adjusted to 10 using 0.1 mol/L NaOH. After mixing for 6 h at 25 °C, the solution was filtered and the resulting powder was washed repeatedly with deionized water to remove unreacted p-BQ, ensuring that the residues were removed as much as possible (until the cleaning solution is colorless and transparent). Next, 50 mL EDA solution (1.8 mM) was added to the activated chitin, and the mixture was allowed to react for 6 h under the same temperature. Deionized water was used to remove unreacted EDA in the same way as unreacted p-BQ was removed. Finally, through acetylation, aminated chitin powder was added to 50% NaOH solution. After reflux reaction at 120 °C for 12 h, the solution was filtered and the resulting AmCS powder was washed until neutral, and then freeze-dried for later use.

### 2.3. Preparation of Folate-Modified Aminated Chitosan Sodium Tripolyphosphate (FA-AmCS-TPP) Nanoparticles

The ion crosslinking method [48,49,50] was used in the preparation of FA-AmCS-TPP nanoparticles. The amount of added raw materials is shown in Table 1. For example, for group S1, 0.05 g AmCS powder was weighed and dissolved in 50 mL acetic acid solution (2%, *v*/*v*), using a 150-mL beaker. The solution was then filtered and the insoluble matter was removed. Then, 10 mg FA was added to 20 mL deionized water and mixed with a few drops (≈0.05 mL) of NH_3_·H_2_O (25 wt %) to fully dissolve the FA. Using the dropper, the whole FA solution was dropped into the prepared 50 mL chitosan solution in a water bath at 55 °C with magnetic stirring at 500 r/min. A mixed solution of AmCS and FA (named AmCS-FA) was obtained. Then, 30 mL TPP solution (containing 10 mg TPP) was trickled dropwise into the solution in the same way. After that, the mixture was stirred for 10 min at the same speed to form a FA-AmCS-TPP nanoparticle water dispersion system. The whole process of FA-AmCS-TPP nanoparticles preparation was conducted in the dark, and the drop rate was maintained at 3 mL/min (about 1 drop/s) using a speed control peristaltic pump (BT100M digital type, Baoding, Shanghai, China).

The other groups (S2–S10) were prepared in a similar manner based on the quantities stated in Table 1. After preparation, the prepared FA-AmCS-TPP nanoparticles water-dispersion systems were stored at 4 °C for later use.

The drug-loaded nanoparticles (Cur/FA-AmCS-TPP) were prepared as described above using Cur (2.0, 5.0, 8.0, 10.0, 15.0, 30.0, and 35.0 mg, respectively) dispersed in 50 mL AmCS, 20 mL FA, or 30 mL TPP solution with a 50-mL conical flask (10 min of 53 Hz ultrasound), using a KeDao SK5210LHC ultrasonic apparatus (Shanghai, China).

**Figure 1:**
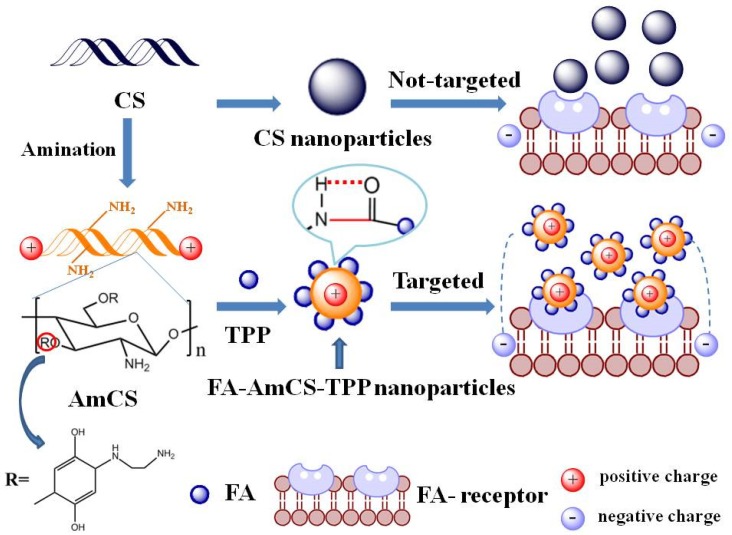
Scheme of the preparation and action mechanism of FA-AmCS-TPP nanoparticles.

The dispersion system was centrifuged at a 4500 r/min for 30 min to obtain precipitates, which were then washed thrice with 30 mL acetic acid solution (0.5%) and 30 mL deionized water, then re-centrifuged for 30 min to obtain the corresponding nanoparticles. The unreacted chain macromolecules (mainly for the AmCS) can be removed through the pinhole membrane filter (Jin Teng, Guangdong, China, material: nylon, d = 25 mm, bore diameter = 0.45 μm). The supernatant was collected and tested for FA and drug-loading levels. Figure 1 shows a schematic diagram of the mechanism of action of FA-AmCS-TPP nanoparticles.

### 2.4. Characterization

#### 2.4.1. Particle Size, Polydispersity Index (PDI), and Zeta (ζ)-Potential Measurement

According to a previous method [51,52,53], the particle size, polydispersity index (PDI) and ζ-potential values of the freshly prepared nanometer water dispersion system were measured by dynamic light scattering using a Zetasizer Nano-ZS90 (Malvern Instruments, Worcestershire, UK). The measurements were performed with a fixed scattering angle of 90° at 25 °C. The water dispersion system was diluted by 5–10 times with deionized water to maintain statistically the count rate at 100–200 kcps. The particle size was expressed as cumulative mean diameter (size, nm). The ζ-potential value was calculated by the instrument using the Smoluchowski model. All measurements were carried out in triplicate.

#### 2.4.2. Determination of FA

The active targeting effect of nanoparticles mainly depends on the content of targeted ligands [54]; thus, FA content in nanoparticles was determined using the following method. The FA-AmCS-TPP nanoparticle water dispersion system was centrifuged at 4500 r/min for 30 min, and the supernatant was put into a 100 mL volumetric flask. Using the S5 group (without FA) as a reference, FA content was determined on an ultraviolet (UV) spectrophotometer at a wavelength of 256 nm. after dilution. FA encapsulation efficiency (EE-FA) was calculated according to Formula (1). The amount of FA added at the time of preparation is denoted as *W_0_*, whereas FA content in the supernatant is represented by *W_F_*.

(1) $$\mathrm{EE}-\mathrm{FA}\left(\%\right)=\frac{W_{0}-W_{F}}{W_{0}}\times 100\%$$

#### 2.4.3. Drug-Loading Studies

The Cur encapsulation efficiency (EE-Cur) and drug-loading capacity (LC-Cur) of the nanoparticles were determined as follows. The amount of Cur added at the time of preparation is denoted as *W_C_*. The prepared drug-carrying nanoparticle dispersion system was centrifuged at 4500 r/min for 15 min, and the supernatant was collected, whereas the precipitates were freeze-dried, weighed, and recorded as *W_m_*. Cur content in the supernatant, which is represented by *W_S_*, was determined by the standard curve method using a UV spectrophotometer at a wavelength of 426 nm. In the determination process, supernatant solution of drug-free nanoparticles prepared under the same conditions was used as a reference. Finally, the EE-Cur and LC-Cur of the nanoparticles were calculated according to Formula (2) and (3), respectively.

(2) $$\mathrm{EE}-\mathrm{Cur}\left(\%\right)=\frac{W_{C}-W_{S}}{W_{C}}\times 100\%$$

(3) $$\mathrm{LC}-\mathrm{Cur}\left(\%\right)=\frac{W_{C}-W_{S}}{W_{m}}\times 100\%$$

#### 2.4.4. In Vitro Release Studies

To test whether the solid nanoparticle powder can be adapted to different pH conditions to achieve good release, we simulated its release in vitro. Freeze-dried nanoparticles (40 mg) were precisely weighed and placed in dialysis bags, and the in vitro release of Cur was studied using the basket method by an intelligent dissolution instrument. The dialysis bag was fixed in the small tank of the basket, and 100 mL phosphatic buffer solution (pH 1.2 or 7.4) was added into the round-bottom beaker. The buffer solutions of pH 1.2 and 7.4 were used to simulate the pH environment of gastric juice and intestinal juice, respectively. The rotation speed was 100 r/min at 37 °C. Next, 2 mL of release solution was collected regularly for content determination using UV, and then the cumulative release rate was calculated. After each collection of release solution, an equivalent amount of buffer was added to the beaker.

#### 2.4.5. Cytotoxicity and Uptake by Tumor Cells

The LS174T cell line was maintained in Dulbecco’s modified Eagle’s medium containing 10% fetal bovine serum, 500 μg /mL penicillin and 100 μg/mL streptomycin and incubated at 37 °C in a humidified incubator (MCO-230AICUVH, Panasonic, Shanghai, China) with 5% CO_2_. Cytotoxicity was determined using a modified 3-(4,5-dimethylthiazol-2-yl)-2,5-diphenyl tetrazolium bromide (MTT) assay [55,56]. Briefly, cells at a density of 7.5 × 10^4^ per well in 200 μL of culture medium were seeded in to 96-well late. These cells were treated with the desired Cur concentration (5.0, 7.5, 10.0, 15.0, 20.0, 25.0, 35.0 and 40.0 μg/mL) of sample (Cur/FA-AmCS-TPP or Cur/AmCS-TPP nanoparticles), and the corresponding concentration of native Cur in aqueous-DMSO (for comparison) for 24 h. Following this, the cells were treated with 50 μL MTT solution (0.5 mg/mL) for 4 h. After incubation, the supernatant was removed, 150 μL DMSO was added, and the OD value at 490 nm was measured with an enzyme-labeled instrument after 10 min of oscillation. The percentage (%) viability was calculated using Formula (4), where OD_s_ is the absorba nce value of the cell hole after drug treatment, and OD_c_ is the absorbance value of the plate hole of the blank control (containing only the culture solution of the same volume).

(4) $$\mathrm{Viability}\;\left(\%\right)=\frac{{\mathrm{OD}}_{s}}{{\mathrm{OD}}_{C}}\times 100\%$$

To evaluate cell uptake of particles, coumarin-6, which has a structure similar to that of Cur, was used as a fluorescent dye and embedded in FA-AmCS-TPP nanoparticles to study the uptake of vectors by colon cancer cells. The method of loading coumarin-6 on the carrier was similar to that of Cur. Specifically, 15.0 mg coumarin-6 was suspended in the prepared 50 mL AmCS solution (2.0 mg/mL), completely mixed, and then successively added to FA solution (20 mL, 0.5 mg/mL) and TPP solution (30 mL, 0.33 mg/mL). For comparison, AmCS-TPP nanoparticles without FA were prepared as described above (except FA solution was not added). Carriers containing coumarin-6 were incubated in a 6-well plate containing 1.2 × 10^6^ per well colon cancer cells for 0.5 h or 1 h, and the results were recorded using a fluorescence-inverted microscope (Leisi, Nikon, Tokyo, Japan).

#### 2.4.6. Other Characterization

The surface morphology of the microgel beads was evaluated by SEM (S-4800; Hitachi Limited Ltd., Tokyo, Japan). For SEM investigation of all water-dispersion system samples (diluted with water approximately five times), the water was evaporated and the system was coated with a thin gold film under vacuum prior to analysis, and these samples were viewed at an accelerating voltage of 20 kV at the appropriate magnification.

TEM images of FA-AmCS-TPP were obtained using Lorentz Transmission Electron Microscope (JEM-2100; JOEL, Tokyo, Japan). An appropriate amount of water dispersion system sample (diluted with water approximately five times) was uniformly dropped onto the copper sample preparation network, and TEM scanning was performed after the water evaporated.

A FT-IR system (Tensor II; Bruker, Bremen, Germany) was used to characterize the functional groups of the materials. In each case, the powdered samples (dilute with water about five times) were placed into a KBr (spectrum pure) pellet, and then the spectra were scanned and recorded from 400 to 4000 cm^−1^ at room temperature (25 °C).

Furthermore, TGA of all samples was performed by employing a TGA instrument (Pyris Diamond TG/DTA; Perkin-Elmer, Waltham, MA, USA) at a heating rate of 10 °C/min from 20 °C to 800 °C under N_2_ atmosphere. The DSC test adopted the following approach: the dry solid sample of 15.0 mg was accurately weighed and put into the aluminum crucible. The whole crucible and the filled samples were added with the crucible cover and pressed into the instrument (DSC 200 F3 Maia^®^, Netzsch, Free State of Bavaria, Germany) for testing. The heating process ranged from 20 °C to 37 °C, the heating rate was 5 °C/min, and the temperature was kept constant for 0.5 h. Nitrogen was selected as the protective gas, and the flow rate was 50 mL/min.

## 3. Results and Discussion

### 3.1. Analysis of Shape and Appearance

**Figure 2:**
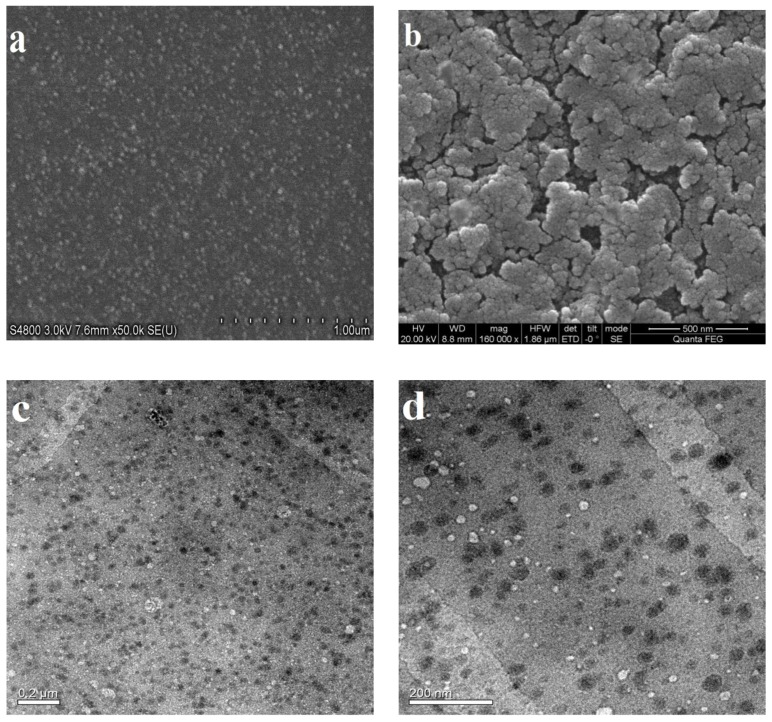
Scanning electron microscopy (SEM) images of FA-AmCS-TPP nanoparticles of component S2 (**a**) and component S10 (**b**). Transmission electron microscopy (TEM) images of FA-AmCS-TPP nanoparticles of component S2 (**c**) and the enlarged image (**d**).

SEM and TEM were employed to observe the morphology of FA-AmCS-TPP nanoparticles, and the results are presented in Figure 2. Figure 2a shows the SEM diagram of the nanoparticles of component S2. For comparison, Figure 2b shows the scanning diagram of the nanoparticles of component S10. It was clearly observed that as the amount of TPP increased, the nanoparticles became agglomerated and multiple-coated, which led to an increase in particle size. We observed the surface morphology of the nanoparticles, and the TEM images of component S2 (Figure 2c,d) showed that the nanoparticles were nearly spherical and uniform in size. This finding suggested that the prepared nanoparticles of component S2 were homogeneous and suitable for further studies.

### 3.2. Fourier Transform Infrared Spectrophotometry (FT-IR)

**Figure 3:**
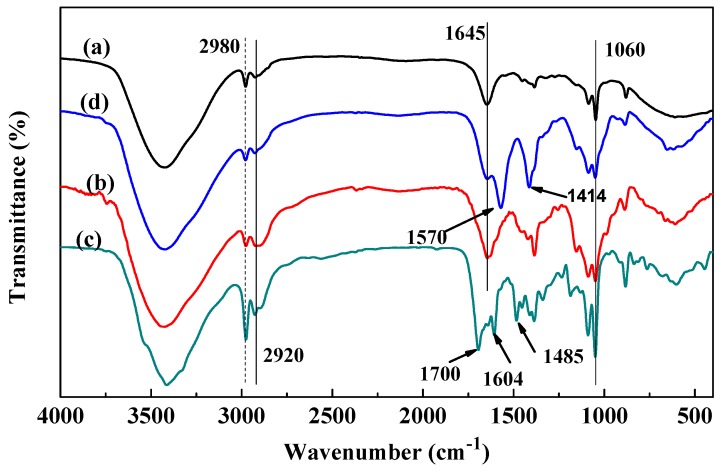
Fourier transform infrared (FT-IR) spectra of (**a**) chitosan, (**b**) AmCS, (**c**) FA, and (**d**) FA-AmCS-TPP nanoparticles.

Figure 3 shows that the infrared spectra of FA-AmCS-TPP nanoparticles (Figure 3d) and AmCS (Figure 3b) were similar, but different from those of FA. This may have occurred because AmCS are the macromolecules constructing the bulk of composite nanoparticles, whereas FA constructs only a small portion of nanoparticles. O–H and N–H stretching vibration peaks appeared at 3600–3200 cm^−1^, and their strong absorption indicated that the nanoparticles contained hydroxyl and carboxyl groups [57]. In addition, because the aromatic ring structure was unique to FA (Figure 3c) in all constituents, the framework stretching vibration peaks of the aromatic ring at 1604 and 1485 cm^−1^ appeared in the polymer [58] (migration to 1570 and 1414 cm^−1^ in the composite nanoparticles), which represented the successful combination of FA and AmCS. The telescopic vibration peak at 1700 cm^−1^ (Figure 3c) can be attributed to the carbonyl group of FA, and its disappearance in Figure 3b indicated that FA and AmCS was bound by an ionic bond, that is, an electrostatic interaction between the carboxyl group of FA and the amino group of AmCS. The stretching vibration of the amino group was retained in the formed nanoparticles, indicating that the surplus amino group was retained in FA-AmCS-TPP, which was conducive to the dispersion of nanoparticles and tumor targeting.

### 3.3. Thermogravimetric Analysis (TGA) and Differential Scanning Calorimetry (DSC)

**Figure 4:**
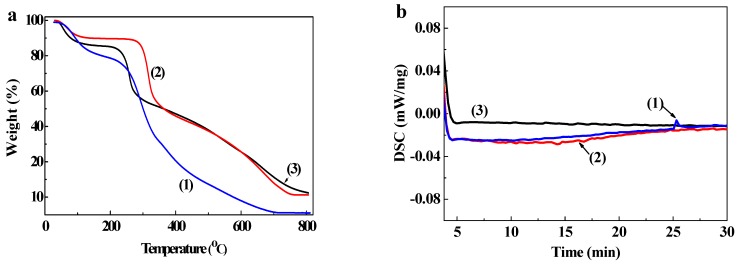
(**a**) Thermogravimetric analysis (TGA) of (1) chitosan, (2) AmCS, and (3) FA-AmCS-TPP nanoparticles; (**b**) DSC of (1) chitosan, (2) AmCS, and (3) FA-AmCS-TPP nanoparticles at constant 37 °C.

Thermogravimetric curves for chitosan, AmCS, and FA-AmCS-TPP nanoparticles are shown in Figure 4a by comparing the curves of chitosan (Figure 4(1)) and AmCS powder (Figure 4(2)), we observed that the thermal stability of AmCS increased significantly after amino modification. When the temperature reached 100 °C, thermogravimetric loss occurred in 10% of samples owing to reticular water loss. AmCS and FA-AmCS-TPP nanoparticles had similar thermogravimetric curves because AmCS was a major component of FA-AmCS-TPP nanoparticles. The weight of FA-AmCS-TPP nanoparticles was constant at above 80% at the temperature range of 80–200 °C because of the degradation of the polymer and hydrogen-bound water that formed the polysaccharide structure of AmCS and chitosan. In addition, a uniform and smooth decline in weight at 250–400 °C was observed in the thermogravimetric curves of chitosan, AmCS, and FA-AmCS-TPP nanoparticles, which is consistent with the general thermal degradation process of chitosan and its relative derivatives [9,59]. According to the literature [47], it is the result of oxidation decomposition of the chitosan skeleton, including the degradation and decomposition of the organic skeletal structure, amino groups, and other functional groups of chitosan. 

DSC analysis results of chitosan powder, AmCS powder, and FA-AmCS-TPP nanoparticles are shown in the Figure 4b. When the temperature rose to 37 °C and was maintained, the DSC of all three samples barely changed in the first 10 min. About 25 min, a small endothermic peak appears on the CS curve, which indicated that the melting process may have occurred. In contrast, this change did not occur in the curve of AmCS powder and FA-AmCS-TPP nanoparticles. Combined with TGA, the results showed that the stability of CS was increased after the amination process, and the stability of composite FA-AmCS-TPP nanoparticles was better than that of single polymer.

The above results suggested that FA-AmCS-TPP nanoparticles would be thermally stable at the physiological temperature of the human body, and can be applied to the physiological system.

### 3.4. Particle Size, PDI, and ζ-Potential

AmCS is a polycation under acidic conditions, whereas TPP is a polyanion in an aqueous solution. The two generate polyelectrolyte through electrostatic interaction, and then they are wound into spherical particles. A solution containing low concentrations of AmCS and TPP is clear and transparent, with almost no condensation. However, a solution containing high concentrations of AmCS and TPP showed large amount of flocculent precipitation. Therefore, the appropriate proportions to obtain the FA-AmCS-TPP nanoparticles need to be explored.

**Figure 5:**
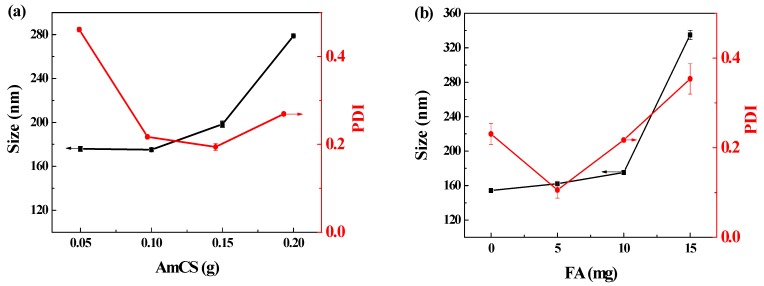

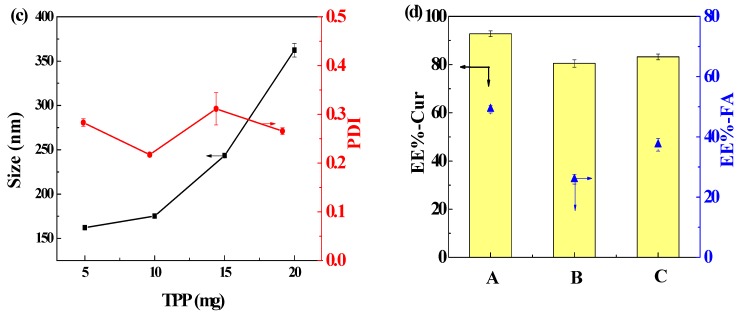
Effect of AmCS dosage (**a**), FA dosage (**b**), and TPP dosage (**c**) on the size and polydispersity index (PDI) of FA-AmCS-TPP nanoparticles. Effect of drug addition method (**d**) on the Cur encapsulation efficiency (EE-Cur, yellow bar graph) and FA encapsulation efficiency (EE-FA, ▲, blue line graph) of Cur/FA-AmCS-TPP nanoparticles: (A) Cur added to the AmCS; (B) Cur added to the FA; (C) Cur added to the TPP.

By analyzing the results shown in Figure 5a, we speculated that when the amount of AmCS in the solution was relatively low (0.05 g), nanoparticles would form quickly after the addition of TPP. However, because the surface potential of the whole particle was still positive, the particles would continue to combine with excessive FA, leading to a high dispersion index (PDI = 0.461 ± 0.005). On the contrary, after the addition of excess AmCS (0.20 g), the nanoparticle size increased to 278.80 ± 0.54 nm. This occurrence can be explained by the possibility that as AmCS concentration increased, the solution viscosity, chance of collision and adhesion, and particle size of the formed nanoparticles also increased. In addition, according the literature [60,61,62], effective diameters of injection nanoparticles were below 200 nm, with PDI ranging from 0.1 to 0.4. The size of FA-AmCS-TPP nanoparticles prepared is 175.2 ± 0.99 nm (PDI = 0.217). In this light, the FA-AmCS-TPP nanoparticles may have potential as injectable agents.

For optimal function, FA content in FA-AmCS-TPP nanoparticles should be appropriate. In this study (Figure 5b), the FA content in the nanoparticles was too small to exert an active targeting role. However, when FA content was elevated to 15.0 mg, the average particle size of the nanoparticles reached 334.90 ± 5.34 nm, as shown in Figure 5b. This may have occurred because the FA content was too high, thereby reducing the binding sites with TPP because too many AmCS were occupying the sites; this led to increased particle size and decreased yield rate. Moreover, because excess FA remained in the solution, it can still bind to positively charged nanoparticles, causing PDI to rise (0.354 ± 0.034).

According to Figure 5c, when the amount of TPP in the system was relatively small (5.0 mg), AmCS concentration was excessive, and the solution was mainly composed of the AmCS-FA complex. The weaker intermolecular interactions were not enough to bend the molecular chain. At that time, the particle size was small (162.03 ± 2.10 nm), but the yield was very low (<10 mg) and this combination is inefficient. However, when the amount of TPP was large, the size of the nanoparticles increased. When the amount of TPP was 20.0 mg, the average particle size of the FA-AmCS-TPP nanoparticles reached 362.23 ± 7.65 nm; this increase may be attributed to the deposition of the nanoparticles, which is consistent with the results in Figure 2b.

Moreover, the ζ-potential test results showed that the surface of FA-AmCS-TPP nanoparticles had a positive charge, and the surface potential of component S2 was large (+42.4 mV) because −NH_2_ transformed into ${-NH}_{3}^{+}$ under acidic conditions, and there was still a large amount of −NH_2_ after the formation of nanoparticles. It is generally accepted that when the absolute value of the surface potential of nanoparticles is greater than 30 mV [63,64], the nanoparticles can exist in a stable state owing to the repulsive effect of electrostatic force. At the same time, particles with positive charges on the surface can easily attract negatively charged particles on the cell membrane, which is beneficial to the distribution of particles in the body after drug administration and is of great significance for the treatment of solid tumors.

### 3.5. Factors Influencing Cur Encapsulation Efficiency (EE-Cur) and FA Encapsulation Efficiency (EE-FA)

In the development of a drug-carrying nanoparticle system, it is necessary to optimize the drug load to ensure effectiveness.

#### 3.5.1. The Effect of Drug Addition Method

In this experiment, drug-loaded nanoparticles (Cur/FA-AmCS-TPP) were prepared by the mixed method in which drugs were wrapped and embedded in the nanoparticles, and some uncoated drugs were also adsorbed on the nanoparticle surface. However, different drug addition methods will lead to different loading effects. Therefore, three methods were used to prepare the drug-carrying nanoparticles, and the loading of Cur was determined. Except for the Cur addition method, the other conditions were consistent with those in Section 2.3, and the amount of raw materials was consistent with S2 in Table 1.

Method A: 5.0 mg Cur was suspended in the prepared 50 mL AmCS solution (2.0 mg/mL), completely mixed, and then successively added to FA solution (20 mL, 0.5 mg/mL) and TPP solution (30 mL, 0.33 mg/mL). 

Method B: 5.0 mg Cur was first mixed with the ammonia solution of FA solution (20 mL, 0.5 mg/mL) and then dribbled into 50 mL AmCS solution (2.0 mg/mL). Finally, TPP solution (30 mL, 0.33 mg/mL) was added to the mixture.

Method C: After mixing with TPP solution (30 mL, 0.33 mg/mL), 5.0 mg Cur was trickled into the AmCS-FA mixed solution.

As shown in Figure 5d, method A achieved the highest EE-Cur (92.80 ± 1.21%) and maintained a great EE-FA (49.22 ± 1.24%) in the case of S2 components. Therefore, method A was adopted to prepare the Cur/FA-AmCS-TPP nanoparticles used in the subsequent studies.

#### 3.5.2. The Effect of Drug Dosage

**Figure 6:**
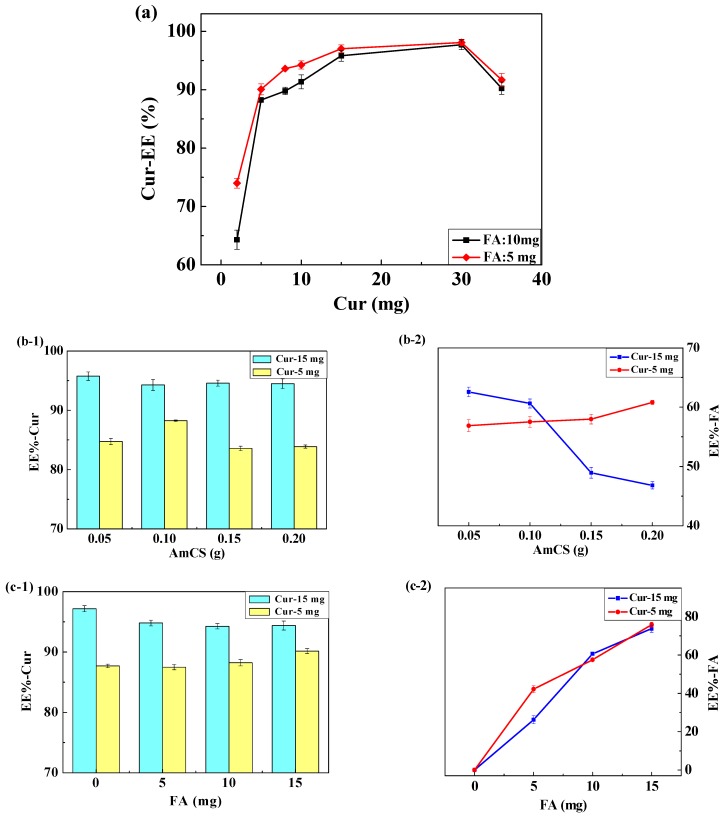

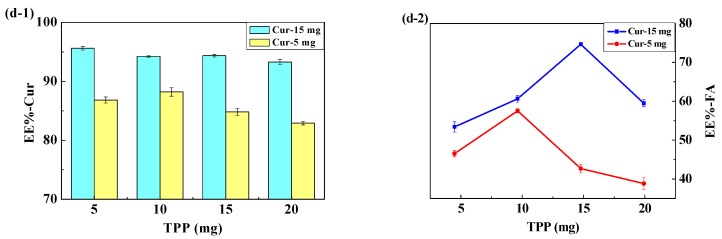
Effect of Cur dosage (**a**), AmCS dosage (**b**), FA dosage (**c**), and TPP dosage (**d**) on the EE-Cur (bar graph) and EE-FA (line graph) of FA-AmCS-TPP nanoparticles. Symbols: Cur 15.0 mg (■); Cur 5.0 mg (●).

Addition of Cur at different amounts can affect the EE-Cur of the nanoparticles. As shown in Figure 6a and Table 2, as the amount of Cur increased with that of the nanoparticle remaining almost constant, the EE-Cur increased first and then decreased. At low amounts of added Cur during preparation, within a certain range, with an increase in the amount of drugs added, the chance of drug loading and EE-Cur increased. With Cur dosage increased from 2.0 mg to 30.0 mg and FA was added at 10.0 mg, the EE-Cur increased from 64.30 ± 1.65% to 97.72 ± 0.84%. The same trend occurred when the amount of FA added was decreased to 5.0 mg. However, since the encapsulation capacity of the carrier was constant, upon addition of too much Cur during preparation, the embedding and adsorption of drugs reached a saturation and the excess Cur was not encapsulated, leading to a decrease in the EE-Cur. As the dosage of Cur increased from 30.0 to 35.0 mg, the EE-Cur decreased to 90.25 ± 1.08% and the particle size increased simultaneously, which was not desirable. Considering these experimental results, it is important to use optimal Cur amount to reduce Cur wastage during preparation and improve the efficiency of drug loading, and thereby ensure that the FA-AmCS-TPP nanoparticles have adequate loaded drug; thus, Cur dosages of 5.0 mg and 15.0 mg were selected for the subsequent studies of the influence of other factors on EE-Cur.

#### 3.5.3. The Effect of Material Concentrations

As shown in Figure 6b, when the amount of Cur was 15.0 mg, there was no significant difference in the EE-Cur of AmCS at different proportions, which indicated that an AmCS amount of greater than 0.05 g was sufficient to load 15.0 mg of Cur and that AmCS tended to combine with the drug. However, with increased amounts of AmCS, the limited TPP can only convert part of the AmCS into nanoparticles, while the remaining AmCS remain floating in solution and prefer to combine with FA, resulting in a decreasing trend of EE-FA. On the contrary, small amount of Cur (5.0 mg) had little effect on the binding of AmCS with FA; thus, the difference in EE-FA was not significant. At Cur amount of 5.0 mg, AmCS increased from 0.05 g to 0.20 g, whereas EE-Cur first increased and then decreased; this may have occurred because the increase in AmCS concentration led to increased encapsulation opportunity, and subsequently, the AmCS without formed microspheres contained more Cur.

As shown in Figure 6c, as FA dosage increased, EE-FA presented an increasing trend, regardless of the amount of Cur. This suggested that AmCS had strong ability to bind to FA, but too much FA would lead to a decrease in the positive charge on the surface of FA-AmCS nanoparticles, which would not be conducive to their binding to tumor cells. Moreover, the effect of EE-Cur on FA was not significant, consistent with the result shown in Figure 6a.

As shown in Figure 6d, as TPP increased (5.0 mg to 10.0 mg), EE-FA also increased (46.51 ± 0.79% to 57.52 ± 0.52%); this can be attributed to the fact that TPP encapsulates more AmCS into the nanoparticles. The increase in the dosage of Cur to 15.0 mg showed the same trend. As TPP amount in the system increased from 5.0 mg to 15.0 mg, the EE-FA increased from 53.40 ± 1.31% to 74.65 ± 0.43%. However, when the dosage of Cur was 15.0 mg, TPP occupied more sites of AmCS owing to an increase in TPP amount, causing faster formation of nanoparticles and decreased EE-FA. When the dosage of Cur was 5.0 mg, the EE-Cur showed the same trend, suggesting that Cur increase was not the main factor, compared with TPP increase. In other words, AmCS had a stronger affinity for TPP than for Cur.

To sum up, when the Cur adding amount is 15.0 mg and the AmCS is 0.10 g, the corresponding FA amount is 10.0 mg, and the TPP is 10.0 mg, the EE-Cur is 94.26 ± 0.91%, and the LC-Cur (29.5 ± 0.62%) and EE-FA (60.62 ± 0.76%) also maintain a relatively high level.

### 3.6. In Vitro Release Studies of Cur/FA-AmCS-TPP Nanoparticles

**Figure 7:**
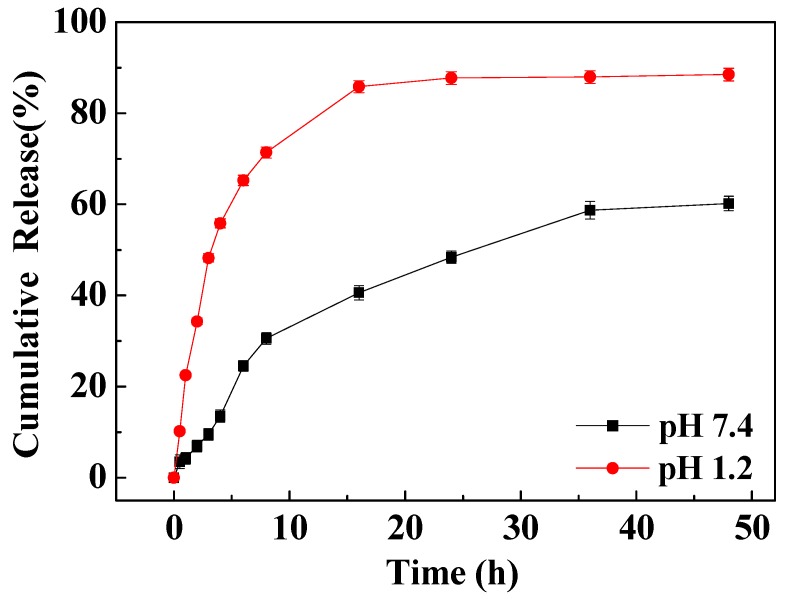
Cur release profiles of Cur/FA-AmCS-TPP nanoparticles in SGF (pH 1.2) and SCF (pH 7.4).

Figure 7 shows that FA-AmCS-TPP nanoparticles exhibited slow and controlled release of Cur in the artificial body fluid (simulated colonic fluid (SCF); pH 7.4). Within the first few hours, Cur was released rapidly, with a cumulative release rate of 3.51% within 0.5 h and 7.52% within 3 h. In the middle stage (after 3–24 h) the release was indirect and near-uniform and the cumulative release within 24 h was nearly 50%, indicating that this release profile was caused by the diffusion of the Cur wrapped in the nanoparticles through the pores on the nanoparticle surface. The release of Cur was very slow at the later stage (after 36 h). The cumulative release rate at 48 h was 56.2%, and a considerable amount of Cur remained in the nanoparticles and could not be released, indicating that Cur was tightly encapsulated in the nanoparticles by AmCS molecules and TPP. Thus, the release required the dissolution of nanoparticles or degradation of AmCS molecules. 

In simulated gastric fluid (SGF; pH 1.2), the cumulative release rate of composite nanoparticles (FA-AmCS-TPP) was relatively high because AmCS tends to dissolve in acid. In the first 8 h, the release rate of Cur was rapid, reaching 48.2% within 3 h; this may allow a rapid drug treatment. After 24 h, the cumulative release rate reached 87.7% and remained stable, suggesting that a significant amount of Cur remained in the matrix and were not released. This may be due to the interaction between the large number of amine groups on the AmCS and the carbonyl group of Cur, which blocks its rapid release.

The release profile of Cur/FA-AmCS-TPP nanoparticles indicated that the dissolution of FA-AmCS-TPP nanoparticles or degradation of AmCS molecules was extremely slow in the absence of enzyme. Therefore, the release of drugs was mainly dependent on the slow dissolution of drugs, causing a very slow release rate of Cur.

### 3.7. Cytotoxicity and Uptake by Tumor Cells

**Figure 8:**
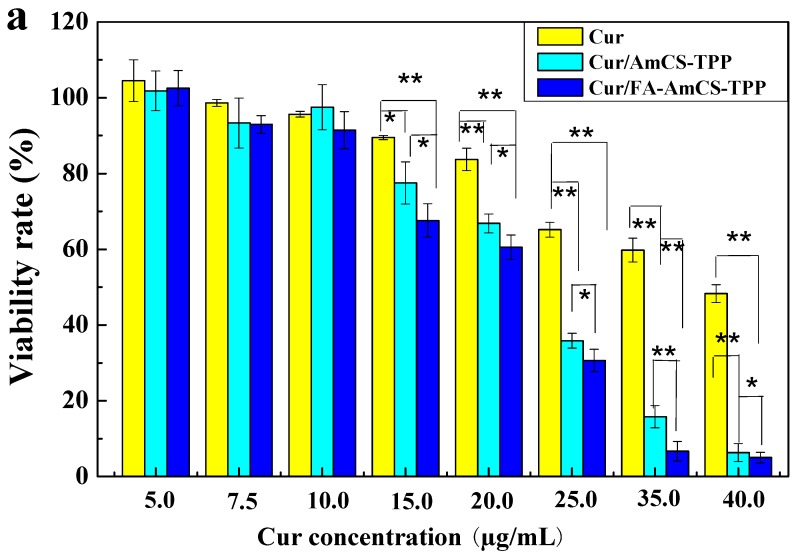

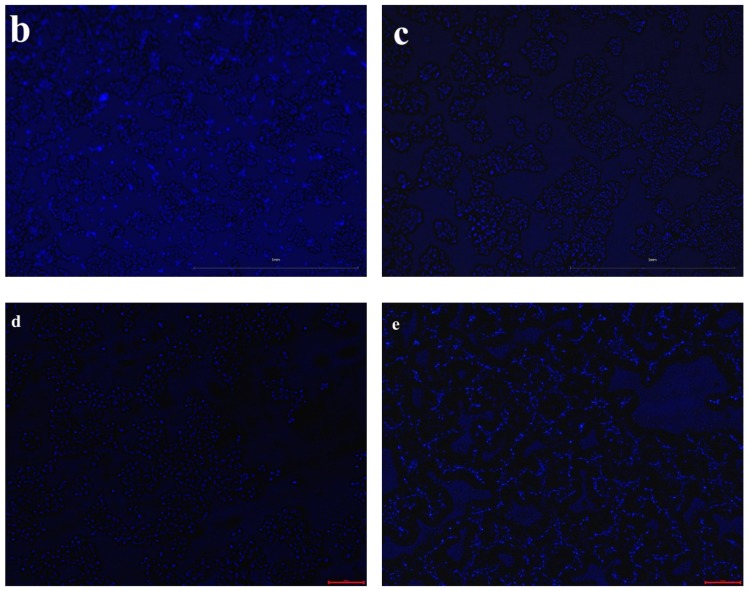
(**a**) Viability rate of LS174T colon cancer cell after treating with Cur (**■**), Cur/AmCS-TPP (**■**) and Cur/FA-AmCS-TPP (■) for 24 h (** *p* < 0.01, * *p* < 0.05, no labeling was not significant); fluorescence microscopic images of LS174T for coumarin-6 loaded AmCS-TPP for 0.5 h (**b**) and 1 h (**d**); fluorescence microscopic images of coumarin-6 loaded FA-AmCS-TPP for 0.5 h (**c**) and 1 h (**e**).

The viability rate of LS174T cells after treating with Cur, Cur/AmCS-TPP, and Cur/FA-AmCS-TPP is shown in Figure 8a. The results showed that the cytotoxicity of each drug group was concentration-dependent, and the cell viability rate decreased with an increase in the drug concentration. In addition, when treated with Cur alone, the cell viability rate decreased, but remained above 60% at the drug concentration of 25.0 μg/mL. Cells treated with nanocarriers (Cur/AmCS-TPP and Cur/FA-AmCS-TPP) showed a viability rate of less than 40% when the concentration reached 25.0 μg/mL. This indicates that carrying Cur on nanocarriers is more likely to cause cytotoxicity. Compared with Cur/AmCS-TPP, Cur/FA-AmCS-TPP exerted a higher cytotoxicity on the colon cancer cells. This is due to the presence of FA, because of which the uptake of Cur/FA-AmCS-TPP nanoparticles by tumor cells was significantly enhanced, resulting in increased cytotoxicity.

As shown in Figure 8b,c, fluorescence microscopy indicated that when cancer cells were treated with coumarin-6 loaded nanoparticles within the same culture time (0.5 h), coumarin-6/AmCS-TPP nanoparticles were more likely to dissociate around cells and less likely to enter into cells, while coumarin-6/FA-AmCS-TPP nanoparticles were more likely to be taken up by cells. When the incubation time reached 1 h, fluorescence was observed in cells, as shown in Figure 8d,e, and fluorescence of the FA-AmCS-TPP nanoparticle group was more significant. The result intuitively shows that the uptake of FA-AmCS-TPP nanoparticles by tumor cells was significantly higher than that of AmCS-TPP nanoparticles. This confirms that the introduction of FA into the carriers can increase the uptake rate by cancer cells, which is consistent with previous reports [65,66,67,68,69].

## 4. Conclusions

Cur-loaded FA-AmCS-TPP nanoparticles were prepared by the ion-crosslinking method. The average particle size was approximately 175 nm, with round and uniform shape, surface potential of +42.4 mV, as well as good particle size stability. The non-toxic and non-biological risk FA-AmCS-TPP nanoparticles prepared by simple ion crosslinking have great potential for carrying Cur. The 94.3% encapsulation rate of Cur was excellent and satisfactory (Cur: 15.0 mg; AmCS: 0.10 g, FA: 10.0 mg, TPP:10.0 mg). At pH 7.4, the Cur/FA-AmCS-TPP nanoparticles showed slow and controlled release of Cur. In particular, the cumulative release rate at 48 h was 56.2%, leaving a substantial amount of Cur in the nanoparticles. Considering the size of particle size (175.2 ± 0.99 nm) and the amount of FA bound to the nanoparticles, we suspected that the nanoparticles will show good tumor-targeting effect and have potential as injectable agents. This was also confirmed by cytotoxicity and uptake experiments. In summary, the FA-AmCS-TPP nanoparticle system is suitable to carry fat-soluble drugs to the tumor tissues and release the drug effectively.

## Figures and Tables

**Table 1:** Quantity of the reactants used in the preparation of folate-modified aminated chitosan sodium tripolyphosphate (FA-AmCS-TPP) nanoparticles.

Samples	AmCS (g)	FA (mg)	TPP (mg)
S1	0.05	10	10
S2	0.10	10	10
S3	0.15	10	10
S4	0.20	10	10
S5	0.10	0	10
S6	0.10	5	10
S7	0.10	15	10
S8	0.10	10	5
S9	0.10	10	15
S10	0.10	10	20

**Table 2:** Effect of Cur dosage on the EE-Cur of FA-AmCS-TPP nanoparticles.

Groups	Cur (mg)	EE-Cur (%)	
		FA = 5 mg	FA = 10 mg
1	2	73.99 ± 0.84	64.30 ± 1.65
2	5	90.06 ± 0.93	88.23 ± 0.25
3	8	93.34 ± 0.14	89.79 ± 0.64
4	10	94.25 ± 0.68	91.36 ± 1.21
5	15	97.02 ± 0.65	95.82 ± 0.94
6	30	98.10 ± 0.55	97.71 ± 0.84
7	35	91.68 ± 1.11	90.25 ± 1.08
